# The Community–Environment–Health Nexus: Connecting Community-Led Climate Action to Health Co-Benefits

**DOI:** 10.3390/ijerph23070844

**Published:** 2026-06-27

**Authors:** Charlotte Wendelboe-Nelson, Kaz Lyon, Erica Alex, Stephen Malden, Andrew J. Williams, Catharine Ward Thompson

**Affiliations:** 1 OPENspace Research Centre, School of Architecture and Landscape Architecture, Edinburgh College of Art, University of Edinburgh, Edinburgh EH3 9DF, UK; c.ward-thompson@ed.ac.uk; 2 Edinburgh Communities Climate Action Network (ECCAN), Edinburgh EH1 3LH, UK; kaz@eccan.scot (K.L.); erika@eccan.scot (E.A.); 3 Scottish Collaboration for Public Health Research and Policy, School of Health in Social Sciences, University of Edinburgh, Edinburgh EH1 2QL, UK; smalden@ed.ac.uk (S.M.); andrew.j.williams@ed.ac.uk (A.J.W.)

**Keywords:** green space, blue space, nature contact, climate action, health co-benefits, urban health, community-led interventions

## Abstract

**Highlights:**

**Public health relevance—how does this work relate to a public health issue?**

- Cities face climate and population-health pressures together, yet the two are usually addressed through separate programmes.
- Community-led climate action routinely brings people into green and blue spaces, an exposure linked to better mental and physical health.

**Public health significance—why is this work of significance to public health?**

- Across 32 climate-focused community projects, health and wellbeing benefits emerged as unplanned secondary outcomes even though health was never a project objective.
- Nature contact tracked closely with these benefits. Projects with high green or blue space engagement reported health gains more often than indoor or infrastructure projects, and even brief outdoor contact outperformed longer indoor programmes.

**Public health implications—what are the key implications or messages for practitioners, policy makers and/or researchers in public health?**

- Funders and commissioners can design climate initiatives for dual environmental and health returns by prioritising green and blue space engagement and linking them to routes such as green social prescribing.
- The proposed Community–Environment–Health Nexus (CEHN) framework gives researchers a testable account of how community agency and environmental engagement combine to generate health co-benefits, setting an agenda for prospective evaluation.

**Abstract:**

Background: Climate action initiatives primarily target environmental outcomes, but their potential for health co-benefits through green and blue space contact remains understudied. Understanding these secondary outcomes could inform dual-purpose programming addressing climate and health challenges. Methods: This retrospective analysis examined 32 community-led climate action projects funded through Edinburgh Communities Climate Action Network (ECCAN) and their GreenLight programme (2024–2025). Projects spanned energy, transport, food, circular economy, and green space themes, with no formal health evaluation framework. Data were extracted from final reports covering 3195 direct participants. Results: Despite health not being a primary objective, 21 of 32 projects (66%) reported secondary health and wellbeing outcomes. Projects involving direct nature contact showed the strongest co-benefits: nine of ten high-nature-contact projects reported health benefits, compared to two of eight low-nature-contact projects. Notable outcomes included 94% of participants reporting increased willingness to engage in future community activities and 76% reporting mental wellbeing improvement following community garden workshops. Blue space engagement demonstrated particular significance for mental health and social cohesion. Conclusions: Green and blue space engagement in climate action projects consistently produces secondary health outcomes. We propose the Community–Environment–Health Nexus (CEHN) framework to understand how community-led environmental action generates synergistic health co-benefits.

## 1. Introduction

Community-led climate action has expanded rapidly as a vehicle for urban environmental change, with growing recognition that such initiatives may generate benefits beyond their primary environmental objectives. Whilst health co-benefits of climate action are acknowledged in major international frameworks [1,2], systematic empirical evaluation of health as a secondary outcome of community-led initiatives remains limited [3,4].

The broader literature on health co-benefits of climate action has tended to focus on large-scale policy interventions, such as active travel infrastructure, air quality improvements, and dietary transitions, rather than on community-led initiatives [5,6]. Emerging evidence suggests this potential is particularly significant when interventions involve contact with green and blue spaces [3,4], yet the mechanisms by which such co-benefits arise, and the conditions under which they are most likely to manifest, remain poorly understood. Similarly, most urban green space and health research examines formal green space interventions or explicit health programmes [7,8], leaving a gap in knowledge regarding health as an unintended secondary outcome of climate-focused community action [9,10].

Green spaces, such as parks, gardens, woodlands, and urban vegetation, have been extensively studied for their physical and mental health benefits [11], with robust evidence for effects including stress reduction, improved mood, enhanced physical activity, and strengthened social cohesion [12,13,14]. Blue spaces, such as rivers, canals, coastal areas, and constructed water features, show similar associations with improved wellbeing, stress reduction, and social connection [15]. However, little is known about how these benefits manifest when nature contact occurs incidentally, through climate action, rather than through recreation, therapeutic programmes, or explicit health interventions, representing a significant gap given the rapid growth of community-led climate action as a vehicle for urban environmental change.

The question of what constitutes a health co-benefit of climate action, and how such co-benefits can be reliably generated, has become increasingly relevant to urban policy and public health practice. As cities seek to respond simultaneously to the climate emergency and persistent health inequalities, there is growing interest in identifying interventions capable of delivering dual benefits: reducing harmful emissions whilst improving population health outcomes [5,6]. Community-led climate action represents a particularly promising, if under-examined, arena in this regard, as such initiatives are characterised by local mobilisation, sustained peer engagement, and the creation of conditions that may facilitate incidental nature contact, all factors associated with positive health outcomes in other contexts [12,16].

The Edinburgh Communities Climate Action Network (ECCAN) GreenLight programme (2024–2025) provides a valuable opportunity to examine these questions under conditions of everyday practice. The programme funded 32 diverse climate projects spanning energy/retrofit, transport/active travel, food/growing, circular economy/waste, and green space/biodiversity, with varying degrees of green and blue space engagement. Importantly, projects were selected on the basis of climate action criteria alone, with no health objectives embedded in their design or delivery.

Health outcomes, where they occurred, therefore emerged organically in conditions unaffected by researcher presence, formal evaluation frameworks, or the demand characteristics that may accompany explicitly health-focused interventions. This characteristic of the dataset provides a rare opportunity to examine health co-benefits as they arise in practice, free from the selection biases and observer effects that may influence findings from dedicated health programmes. Edinburgh’s urban geography, combining post-industrial neighbourhoods with accessible green space and waterways, and its active community infrastructure, further make it a productive and instructive context in which to examine these questions.

This study examines the potential for health and wellbeing benefits as secondary outcomes across these climate-focused projects, with particular focus on how green space, blue space, and nature contact may act as mediating or moderating pathways for any such benefits. In doing so, it aims to contribute to three intersecting areas of scholarship: the empirical literature on nature-based health benefits [11,12]; the policy literature on health co-benefits of climate action [5,6]; and emerging theoretical understanding of how community-led environmental action generates wellbeing outcomes.

### Research Objectives

This study addresses two research objectives:

Objective 1: Retrospective Health Outcome Analysis. To systematically identify and analyse health and wellbeing outcomes that emerged as secondary benefits from community-led climate action projects, examining the prevalence, nature, and characteristics of these unintended health co-benefits across diverse intervention types.

Objective 2: Green and Blue Space Pathway Analysis. To examine the role of green space, blue space, and nature contact in mediating health co-benefits from climate action.

These objectives collectively aim to advance understanding of nature-mediated health co-benefits from climate action interventions and to develop a provisional theoretical framework explaining the synergistic relationships and potential pathways observed.

## 2. Materials and Methods

**Figure 1:**
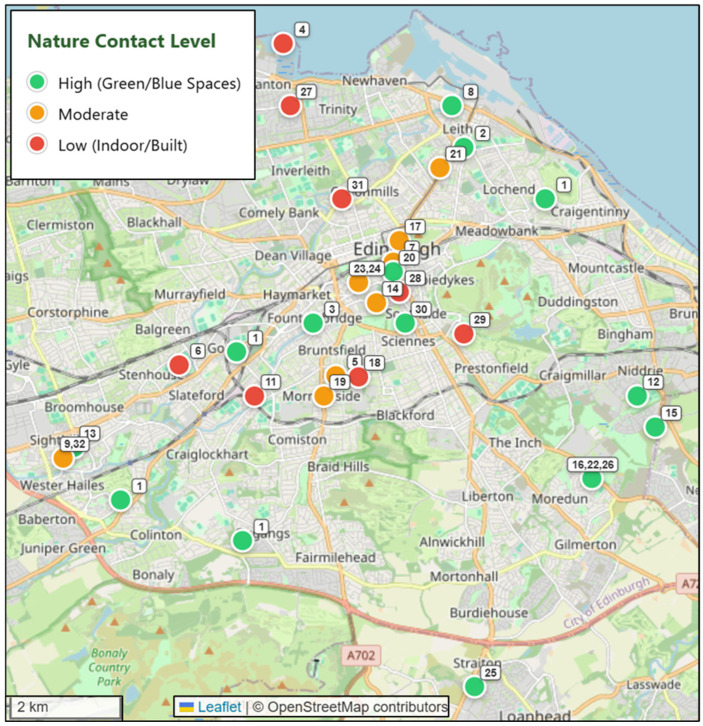
Geographic distribution of ECCAN GreenLight projects (2024–2025). Map of Edinburgh showing locations of 32 community-led climate action projects, represented by 30 markers (numbers correspond to projects listed in Appendix A). Some locations host multiple projects and display multiple project numbers (e.g., Gracemount Community Walled Garden hosts projects #16, #22, and #26). Edible Estates (#1) operated across four neighbourhoods. Markers at identical locations have been slightly offset for visibility.

This retrospective analysis utilised final project reports from all 32 GreenLight-funded projects (March–April 2025) (Figure 1 and Appendix A). Projects were categorised by their degree of green and blue space engagement to examine the relationship between nature contact and health outcomes. High-nature-contact projects involved sustained direct immersion in outdoor natural environments—including woodland, gardens, parks, or waterways—where participants were physically present in and actively engaging with green or blue space throughout the activity. Moderate-nature-contact projects included indoor activities that incorporated natural materials, growing or gardening elements, or nature-themed content, where nature was present but participants were not fully immersed in an outdoor natural environment. Low-nature-contact projects primarily focused on indoor or built environment activities with minimal natural elements.

This retrospective analysis was the methodological approach most appropriate to the research questions for three reasons. First, the research questions are retrospective by design: the GreenLight projects were selected on climate action criteria, meaning any health outcomes that emerged were genuinely secondary and unplanned. A prospective design would have required health measurement to be built into project delivery from the outset, which would have embedded health framing into a context deliberately free of it, and risked observer effects shaping the outcomes we sought to examine. Second, secondary analysis of implementer reports is an increasingly recognised approach in community health research for examining unintended programme outcomes, particularly where data were generated through practice rather than research [17,18,19]. Masefield et al. (2020) [19] identify it as an important but largely underused opportunity for health policy and systems research, and this study contributes to that emerging evidence base. This approach allows authentic outcomes to be surfaced from real-world settings while remaining transparent about the analytical limitations of post hoc interpretation. Third, the diversity of project types—spanning energy, transport, food, circular economy, and green space themes—made systematic prospective measurement impractical within a single evaluation framework. This retrospective analysis was therefore designed as the first stage of a two-stage research programme: the inductive cross-project pattern analysis reported here is intended to underpin the CEHN framework, which is now being tested through a prospective evaluation currently underway. The retrospective inductive approach enabled cross-project pattern analysis whilst preserving the integrity of each project’s primary climate action mandate.

Edinburgh’s green and blue space assets engaged by projects included community gardens and growing spaces such as Leith Community Croft, Gracemount Community Walled Garden, and Meadows Community Garden (Figure 1). Urban woodlands and parks featured prominently, including Craigmillar Castle Park, local orchards, and Frogston wildlife garden. Blue spaces encompassed the Water of Leith and Union Canal, while urban nature corridors included the Roseburn Path and various cycling routes through green networks.

Ethics approval was obtained from University of Edinburgh’s ‘Edinburgh College of Art’ Ethics Committee to undertake this research. The analysis involved secondary data in the form of project reports that were already in the public domain or shared with explicit consent from participating organisations. All project reports were anonymised during analysis, and any identifying details of individual participants mentioned in reports were removed or generalised to protect privacy. Specifically, participant names were replaced with pseudonyms or role descriptors (e.g., “community researcher,” “parent participant”), identifying locations beyond neighbourhood level were omitted, and unique personal circumstances that could enable identification were described in general terms while preserving the essence of reported outcomes.

The reports followed a standardised template requiring organisations to describe their project activities, quantify impacts and beneficiaries, reflect on learnings, and share any climate-related stories from participants. Rather than applying predetermined wellbeing categories, we adopted an emergent, inductive approach to identify any outcomes that project leaders reported as beneficial to participants’ overall quality of life, functioning, or flourishing. This inclusive approach was necessary given that the reports were not designed as wellbeing evaluations and used varied terminology to describe participant benefits. The primary author conducted initial coding of all reports. Recognising that initial single-coder classification carries inherent interpretive risk, all codes were subsequently reviewed independently by two ECCAN co-authors (K.L. and E.A.) who had direct operational knowledge of each project; any discrepancies or contested classifications were resolved through iterative group discussion until consensus was reached. Instances of interpretive difficulty were flagged and documented explicitly, including the rationale for contested decisions, to support transparency and replicability. Examples included distinguishing participant satisfaction from sustained wellbeing improvement, and determining whether reported social connection constituted evidence of reduced psychological isolation.

We coded all mentions of outcomes showing wellbeing benefit to participants, acknowledging that our analysis reflects what project staff chose to observe and report rather than systematic wellbeing measurement. Through this iterative coding process, five main categories of wellbeing outcomes emerged: physical health, mental health, social connection, personal development, and sense of agency or empowerment. These categories align broadly with World Health Organization (WHO) definitions of health and wellbeing, though they emerged organically from the data rather than being imposed as an a priori framework. For the purposes of this analysis, ‘wellbeing’ is used as a broader umbrella term encompassing subjective quality of life and positive functioning, whilst ‘health’ refers to more specific functional states, such as physical or mental, consistent with WHO definitions. Satisfaction ratings were treated as proxies for wellbeing only where they were embedded in reports describing sustained positive participant experience, rather than immediate hedonic response to a single activity. Environmental psychology constructs such as nature connectedness, place attachment, and perceived restorativeness were coded as contributing to wellbeing outcomes where project reports described these in relation to participant flourishing, rather than treating them as independent outcome categories. Biophysical health outcomes (e.g., physical activity, active travel) were coded where explicitly described in project reports, but are treated as indicative rather than confirmed, given the absence of clinical or objective measurement. These observations inform the direct biophysical pathway in the CEHN framework as theoretically grounded proxies, pending validation through prospective studies with appropriate measurement instruments.

### Data Extraction

The data consisted of structured final reports submitted by project leaders to the funding organisation (ECCAN) at the completion of their community climate action projects. The GreenLight programme ran from August 2024 (application opening) to March 2025 (project completion deadline), with awards announced in September 2024. Project durations varied considerably, ranging from intensive workshop series lasting less than one week to sustained initiatives spanning the full 6–7 month funding period, with most projects implementing activities between November 2024 and March 2025.

Across the 32 projects, a total of 3195 direct participants were reported. Participant numbers per project varied considerably, from small group interventions (fewer than 20 participants, e.g., Youth Vision’s programme with 11 vulnerable young people) to large community events (e.g., Bridgend Farmhouse’s apple pressing event with 266 participants) or school-based programmes reaching over 2000 (e.g., Parents for Future Scotland, which reached 2635 children across 20 schools). This heterogeneity reflects the diversity of project types and delivery models funded under GreenLight, and means that aggregate participant counts should be interpreted as indicative of reach rather than as a basis for per capita comparison across projects. Individual-level demographic data (age, gender, ethnicity, socioeconomic status) were not systematically collected or reported across all projects; where available, these are noted in individual project descriptions but could not be used for subgroup analysis across the sample.

We identified and coded explicit mentions of outdoor activities, time spent in green or blue spaces, and direct engagement with natural environments. Activities were categorised by setting (woodland, parks, gardens, waterways) and type (maintenance, exploration, skill building, observation). Projects were then classified into three intensity categories based on reported time spent in outdoor nature contact: high level of contact (7+ h per week), moderate intensity (2–6 h per week), and low intensity (less than 2 h per week or primarily indoor activities with nature themes). We systematically recorded programme length, session frequency, and nature contact level where reported, noting whether activities were one-off events, short-term series, or sustained long-term programmes. These thresholds were developed inductively through a pilot review of five project reports prior to full coding, reflecting the observed range of nature contact intensity across GreenLight projects rather than pre-existing external benchmarks. Where reported contact hours were ambiguous or not directly stated, classification was based on the weight of evidence from activity descriptions, session formats, and programme schedules. Borderline cases, for example a project delivering primarily indoor workshops that also included regular outdoor excursions, were discussed between the primary author and the two validation reviewers before a final classification was agreed collectively. The primary basis for classification was the setting and duration of participant activities rather than the thematic content of the project, so that a project focused on climate education delivered entirely indoors would be classified as low nature contact regardless of its subject matter. For wellbeing outcomes analysis, we coded all mentions of health, wellbeing, confidence, social connection, or personal development outcomes reported by participants, facilitators, or observers. We recorded these outcomes as reported, recognising that they may or may not be directly attributable to nature contact, as distinct from social activities, skill development, or other programme elements. Therefore, when comparing reported outcomes across the three levels of nature contact categories, the findings reflect what participants reported experiencing, rather than what they attributed to any specific programme element.

We systematically coded the physical setting where activities took place based on project descriptions—whether projects operated in indoor spaces, outdoor natural environments, or mixed settings—and noted specific environmental features mentioned such as sensory gardens, woodland areas, or water features. Where project reports explicitly mentioned participants’ connections to these settings, we also identified expressions of attachment to specific places (e.g., “*I feel connected to this woodland*”), environmental stewardship behaviours (e.g., litter-picking, tree planting, habitat restoration), and observations of environmental changes (e.g., noting earlier spring blossoms, declining bee populations, seasonal flooding patterns) as indicators of place-based attachment development.

## 3. Results

### 3.1. Project Classification and Health Outcome Analysis

Table 1 provides a comprehensive breakdown of all projects, their nature contact categorisation, and specific health outcomes reported.

Our analysis adopted an inclusive approach, as described above, given that the original project reports were not designed as systematic wellbeing evaluations and used varied terminology to describe participant experiences. We classified outcomes such as satisfaction ratings (e.g., Earth in Common’s 4.93/5 participant satisfaction), social connection measures (feeling more connected to community), confidence building (teachers noting reduced school refusal and increased peer interaction), and expressions of reduced isolation or anxiety as indicators of mental health benefit. The consistency of wellbeing themes across diverse projects, particularly the clear pattern linking the nature contact level with health outcome reporting, suggests these findings represent meaningful, if preliminary, evidence of health co-benefits from community-led climate action.

### 3.2. Nature Contact Level

The systematic analysis revealed a clear distribution across nature contact categories, with 14 projects (43.8%) classified as moderate nature contact, 10 projects (31.3%) as high nature contact, and eight projects (25.0%) as low nature contact (Table 1). Health outcome reporting demonstrated a strong relationship with nature contact intensity, with 9 of 10 high-nature-contact projects reporting health benefits compared to 10 of 14 moderate-nature-contact projects (71%) and only two of eight low-nature-contact projects (25%). Overall, 21 of the 32 projects (66%) reported some form of health or wellbeing outcomes, despite health not being a primary objective of the climate-focused funding programme.

Projects with higher levels of nature contact demonstrated meaningfully higher rates of spontaneous health outcome reporting, with high-nature-contact projects showing nearly four times the rate of health benefit reporting compared to low-nature-contact projects. However, factors such as project duration and time spent with participants may also contribute to this pattern (see Section 3.5).

### 3.3. Examples of High-Nature-Contact Projects and Health Outcome Reporting

#### 3.3.1. Direct Green Space Engagement

Earth in Common conducted outdoor workshops at Leith Community Croft, achieving a participant satisfaction of 4.93/5, with spontaneous wellbeing reporting. Participants specifically valued “*meeting new people and working outside in the fresh air*” and finding it “*really lovely to just connect with nature in the city*”. The outdoor setting appeared crucial, with 90.2% enjoying “*the social aspect while working in nature*”.

The project’s climate stories captured direct nature–health connections: “*I pledge to reduce synthetic and toxic materials in my home*” and “*I will shift my thinking to find the beauty in the impermanence in all things. I will do my best to protect people and planet without falling into despair*”.

Youth Vision worked with 11 vulnerable young people across 1.5 acres of farmland including a forest garden and sensory garden. The extended outdoor engagement produced remarkable developmental outcomes. Teachers reported: “*At the start of the year, he would cry every day coming to school and sometimes refuse to get into class. He has grown in confidence and made friends*”.

Children specifically valued the outdoor natural setting: “*It was a good choice to come here. I liked digging and making bridges in the pond/mud*” and “*always learning about nature—it was super-fun and I was super-happy*”. One child reflected: “*I feel sorry that my parents gave me an iPad, because all I want to do when I get home is play on it and watch things on it*”, suggesting nature contact provided a meaningful alternative to screen-based activities.

The Green Team’s 13-week programme in a local wildlife garden demonstrated sustained nature engagement benefits. Working with 16 children aged 5–8 years, the project progressed from initial exploration to conservation tasks including “*building a dead-hedge*”, “*planting of trees and daffodils*”, and “*making pine-cone birdfeeders*”.

Teacher evaluations using seven-point scales showed strong wellbeing outcomes, with enhanced pupil confidence and wellbeing scoring 6/7, increased use of green spaces and appreciation for nature rating 5/7, and enhanced social skills including teamwork and communication also achieving 5/7. Teachers provided the highest possible rating of 7/7 when asked whether they would recommend The Green Team to other schools.

Children’s feedback emphasised nature connection: “(I would like to) *continue building the nature pond, because it’s good for the animals*” and “(I would like to) *continue to be outside more*”.

Edible Estates’ community-led climate adaptation project demonstrated how extended nature-based programming can build both environmental awareness and community capacity. Working across four Edinburgh neighbourhoods, the project trained 13 community researchers in climate adaptation while conducting outdoor workshops and mapping sessions.

The project’s health co-benefits emerged through the capacity-building process itself. One community researcher reported that their experience directly led to securing full-time employment in community organising, crediting their climate research work for their successful application. The 22 organisational partnerships developed with the funding provided communities with expanded social networks and increased local buy-in to environmental planning.

Across 15 community workshops with approximately 110 attendees in total, participants engaged with climate adaptation through place-based outdoor activities including park assessments, green space mapping, and flood risk evaluation. The extended engagement (running over several months) allowed for deeper community relationship building and sustained environmental learning compared to single-session interventions.

##### Community Gardens and Food-Growing Spaces

LGBT Health and Wellbeing held their growing workshop in a community garden setting, achieving positive social and mental health outcomes. The majority of participants (94%) felt more able to participate in their community in future, while 88% felt more connected to their community and 76% reported mental wellbeing improvement. Additionally, over half of participants (53%) felt less isolated following the workshop.

Participant feedback emphasised the outdoor natural setting: “*I so enjoyed observing beauty and the breadth of queerness in nature today and how this reminded me that we, as LGBTQ+ people, are not abnormal or less deserving of respect but are in fact completely natural and like the planet, worth respecting and fighting for*”.

Bridgend Farmhouse used an orchard and farmhouse grounds for their apple pressing event, attracting 266 participants. The natural setting contributed to community connection, “*Such a nice friendly event, my kids had a ball*”, and participants valued working outside and connecting with food production processes.

##### Urban Woodland and Orchard Restoration

Drylaw and Telford Community Association worked in two ‘secret orchards’ with children and adults learning “*how to prune and graft new branches onto the trees*”. The project engaged 36 children from Ferryhill Primary School plus local adults in hands-on orchard care.

The project leader’s climate story revealed deep connection between extended nature contact and climate awareness: “*Over the twenty years I have lived in the area, I have noticed the tree in the neighbouring garden blossom earlier and earlier. This year the tree was in bloom in January, a time when there are few pollinators to distribute the pollen*”.

##### Blue Space Engagement and Health Outcomes

The Meaning Map Project conducted workshops along the Water of Leith, including an “*Outdoor Workshop with Volunteers at the Water of Leith Conservation Trust’s Clean-Up at Coalie Park, Leith*”. Participants created meaning maps documenting their emotional connections to the waterway.

The blue space setting facilitated both individual reflection and community connection. Participants reported feeling “*very accessible and nourishing and I feel topped up!*” and valued “*Having a place to connect with the ones who care*”. The project captured numerous nature memories specifically related to the Water of Leith, including sightings of kingfishers and other wildlife.

Porty Community Energy’s ‘Travel Agents of Change’ exhibition was displayed along the Union Canal, among other locations. While primarily focused on sustainable travel awareness, the waterside setting contributed to community engagement and the project’s success in reaching 101 people at exhibition openings.

### 3.4. Examples of Moderate-Nature-Contact Projects and Health Outcome Reporting

#### 3.4.1. Indoor Nature-Based Activities

Edinburgh & Lothians Regional Equality Council’s (ELREC’s) textile upcycling workshops, while held indoors, incorporated natural materials and nature themes. The ‘Sustainable Rug Weaving Workshops’ used recycled fabrics and emphasised connections to natural processes. Despite indoor settings, these projects generated significant social outcomes: “*Participants have formed long-term friendships through the workshops, which has helped them feel less lonely and isolated*”, which in turn will positively affect wellbeing.

Salisbury Centre’s seed library project involved indoor workshops but had strong connections to growing and natural cycles. Participants reported: “*I thoroughly enjoyed the event and have since implemented some of the things I learned in my own garden!*”, suggesting the nature-focused content generated engagement that extended to outdoor spaces.

#### 3.4.2. Active Travel Through Green and Blue Networks

Bikes for Refugees enabled cycling access through Edinburgh’s green networks, with recipients reporting mental health benefits from outdoor movement through natural settings. One participant noted: “*I ride my bicycle every day. I go to the gym, the market, and for outings. This helps me maintain my overall health as well as my mental well-being…I love the feeling of fresh air on my face*”.

Another participant emphasised the restorative aspect of cycling through urban nature: “*my bike gives me a sense of escape. It allows me to tour around the city while listening to music, which helps with loneliness*” and “*When I have a bad time, I use the bike to go out to get some fresh air and refresh my mind*”.

BANZAI’s cargo bike library enabled families to access Edinburgh’s cycling infrastructure, often through parks and green routes. Parents noted safety and wellbeing benefits: “*This is such a great initiative! As someone who doesn’t drive and has to transport children around town in high traffic areas that aren’t safe for young cyclists this was an ideal solution*”. Improvements in feelings of safety for yourself and your children, and the opening up of opportunities to explore the city and create memories with your family, will inevitably affect feelings of wellbeing.

Parents for Future Scotland’s air pollution programme demonstrated how climate education combined with advocacy can generate health co-benefits across entire school communities. Working with 20 Edinburgh schools, the project reached 2635 children through classroom activities and 132 parents through facilitated workshops.

While primarily focused on air quality awareness, the project’s emphasis on active travel connected families to Edinburgh’s green cycling networks. The programme achieved remarkable engagement outcomes: 96.4% of participating parents reported learning something new about air pollution, while 82.3% indicated increased likelihood of using active travel following the workshops.

The culminating City Chambers event, attended by 74 children, teachers, and parents alongside 15 councillors, created a powerful platform for community advocacy around cleaner air and safer streets. This combination of education, outdoor awareness, and collective action appeared to generate both individual health consciousness and community efficacy for environmental change.

### 3.5. Duration and Intensity of Nature Contact

To further examine the relationship between nature engagement and health outcomes, we conducted a detailed analysis of project duration and the level of contact with nature, focusing specifically on projects with clear green or blue space components. This analysis aimed to determine whether a relationship exists between the length and intensity of nature contact and the likelihood of generating health co-benefits. We categorised all 32 projects by total participant engagement time (regardless of the setting) into three tiers: extended (20+ contact hours across multiple sessions over 8+ weeks), moderate (4–20 contact hours, whether delivered as intensive full-day sessions or spread across 2–7 weeks—both treated as equivalent for the purposes of this analysis), and brief (1–4 contact hours delivered in 1–2 sessions). Projects were also separated by setting: those with direct green/blue space involvement versus those focused primarily on indoor or built environment activities (Table 2). This approach allowed us to examine both the independent effects of duration and the moderating role of natural settings in generating incidental health outcomes from climate action programming. The analysis revealed a clear pattern linking duration and intensity of nature engagement with frequency of reported health outcomes.

Extended-engagement programmes of eight weeks or more demonstrated the strongest health outcomes, with all three projects achieving positive health outcome reporting. These included Youth Vision’s 13-week farmland and edible woodland programme, The Green Team’s wildlife garden sessions, and Earth in Common’s six-month outdoor workshop initiative at Leith Community Croft (Appendix A). These extended programmes generated meaningful behavioural changes, confidence building, sustained community connections, and nature-based skill development.

Moderate-engagement projects, lasting from one full day to seven weeks, achieved 90% health outcome reporting across ten programmes including LGBT Health & Wellbeing’s intensive garden workshop, ongoing bike distribution schemes enabling green route access, seed saving workshops, orchard restoration sessions, and cycling programmes connecting families to green networks. These moderate-engagement projects typically produced nature connection, reduced isolation, outdoor skill development, and community connections through green spaces.

Brief-engagement projects involving one to two sessions achieved 83% health outcome reporting across six initiatives, including single outdoor events like apple pressing, waterside exhibitions, and park assessments, generating positive nature experiences and immediate environmental connections. In contrast, the 13 projects with no clear green or blue space component achieved only 38% health outcome reporting, encompassing indoor workshops, infrastructure projects, and equipment purchases that produced social connections and skill development but demonstrated limited nature-mediated health benefits compared to their outdoor counterparts.

The duration-intensity analysis revealed that both programme duration and the natural setting predict health outcome reporting. Green and blue space projects demonstrated higher health outcome reporting across all duration categories, with 89% of nature-based projects (17/19) reporting the generation of health benefits compared to only 38% of indoor/infrastructure projects (5/13)—a 2.3-fold difference. Within nature-based programming, a consistent pattern also emerged—outcome reporting increased with engagement length, from 83% in brief interventions to 100% in extended programmes. While the absence of robust quantitative measurement means this gradient cannot be characterised as a dose–response relationship, the pattern is suggestive and warrants more systematic investigation in future evaluations.

Notably, even brief nature contact (83%) substantially outperformed longer indoor programmes (38%) in health outcome reporting, suggesting that natural environments offer inherent health-promoting qualities that amplify climate programming outcomes regardless of duration. Among the 13 projects focusing on indoor workshops, infrastructure, or built environment activities, health benefit reporting was limited to basic social connections rather than the wellbeing improvements, confidence building, and transformative behavioural changes reported in nature-based interventions. These findings suggest that while duration matters within nature settings—with extended engagement producing the most transformative reported outcomes—the presence of green or blue space contact may be a critical factor determining whether climate action generates incidental health co-benefits.

### 3.6. Climate Anxiety and Nature-Based Coping

Several projects involving direct nature contact showed evidence of constructive climate anxiety management. Earth in Common participants made nature-connected pledges: “*I will shift my thinking to find the beauty in the impermanence in all things. I will do my best to protect people and planet without falling into despair*”. The combination of climate action with nature contact appeared to provide agency and hope rather than overwhelm, contrasting with projects that discuss climate issues without direct environmental engagement.

### 3.7. Urban Nature Access and Health Equity

Looking at the geographic distribution of projects, there was a notable clustering in areas with limited private green space access, suggesting that community green spaces may be especially valuable for promoting health equity. Of those who attended ELREC’s outdoor-connected workshops, 60% came from ethnic minority backgrounds, while LGBT Health and Wellbeing created inclusive nature access specifically for lesbian, gay, bisexual, transgender, and queer/questioning+ (LGBTQ+) community members, demonstrating how these projects enhanced sociocultural accessibility to green spaces.

Both Edible Estates and Parents for Future Scotland demonstrated targeted approaches to health equity through climate action. Edible Estates specifically worked in areas with limited private green space access, while Parents for Future Scotland’s school-based approach reached families across diverse socioeconomic contexts, suggesting that different programming models can effectively address environmental and health inequities simultaneously.

Economic barriers were also addressed through deliberate programming choices, with Earth in Common noting they “*made sure to have different ticket prices for each event, allowing people to choose a concession price if needed*” and ran “*a free Ostara [spring equinox] celebration*” to ensure that cost would not prevent community members from accessing nature-based projects and their associated health benefits.

## 4. The Community–Environment–Health Nexus (CEHN) Framework

### 4.1. Community–Environment–Health Nexus

**Figure 2:**
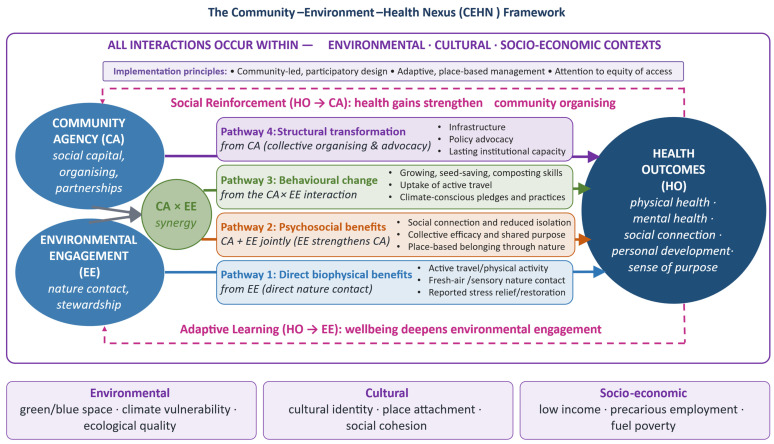
The Community–Environment–Health Nexus (CEHN) framework. The framework was derived inductively from the 32 community-led climate action projects and is informed by the prior literature; the directions shown are proposed rather than statistically tested. Three core components—community agency (CA) and environmental engagement (EE), on the left, and health outcomes (HOs), on the right—are linked by four colour-coded pathways (arrows), each labelled with its origin and illustrative reported outcomes. The four pathways occupy different positions along the results chain (from enabling conditions through intermediate levers to final health outcomes); see Section 4.1.2. Pathway 1, direct biophysical benefits (blue), arises from EE through direct nature contact (e.g., outdoor physical activity and active travel, fresh air and sensory nature contact, reported stress relief and restoration). Pathway 2, psychosocial benefits (orange), arises from CA and EE jointly, with EE strengthening the effect of CA (e.g., social connection and reduced isolation, collective efficacy and shared purpose, place-based belonging through nature). Pathway 3, behavioural change (green), arises from the synergistic CA × EE interaction, shown as the central CA × EE node (e.g., growing, seed saving and composting skills, uptake of active travel, climate-conscious pledges and practices). Pathway 4, structural transformation (purple), arises from CA through collective organising and advocacy (e.g., infrastructure, policy advocacy, lasting institutional capacity). Two feedback loops (pink dashed arrows) sustain the system over time, each returning from HOs to a component: Social Reinforcement (HO → CA), whereby health gains strengthen community organising, and Adaptive Learning (HO → EE), whereby experienced wellbeing deepens environmental engagement. Health outcomes span physical health, mental health, social connection, personal development, and sense of purpose. All interactions are situated within environmental (e.g., green/blue space access, climate vulnerability, ecological quality), cultural (e.g., cultural identity, place attachment, social cohesion), and socioeconomic (e.g., low income, precarious employment, fuel poverty) contexts, and are guided by three implementation principles—community-led, participatory design; adaptive, place-based management; and attention to equity of access [13,14,15]. The CEHN framework draws on three complementary theoretical traditions. Salutogenesis [18] provides the orienting framework: health arises from a sense of coherence—meaning, manageability, and comprehensibility—that community environmental action can strengthen. Social–ecological theory [23,24] recognises that health is shaped not only by individual behaviour but by multiple interacting levels of context, including family relationships, community resources, social policies, and environmental conditions; the framework draws on this to explain why the setting and social context of climate action matter for health outcomes, not just the activities themselves. Finally, two mechanisms explain how environmental engagement specifically contributes: collective efficacy theory [25,26] accounts for the health benefits of community organising and shared agency, whilst the biophilia hypothesis [31,32] explains the independent contribution of nature contact to psychological restoration and wellbeing [12,35].

The patterns observed across GreenLight projects suggest interconnected relationships between community action, environmental engagement, and health outcomes. Figure 2 presents a conceptual framework illustrating these relationships as they emerged from the data. Grounded in systems thinking [20,21], the framework draws on salutogenesis [22], social–ecological theory [23,24], collective efficacy [25,26], attention restoration and stress reduction theory [27,28,29,30], and the biophilia hypothesis [31,32] to explain how health emerges from dynamic interactions between people, place, and collective action [23,33,34]. The four observed pathways through which these mechanisms operate are described in the subsections below.

The framework presented below should be read in direct relation to the two research objectives. Objective 1 is addressed by the cross-project analysis in Section 3.1, Section 3.2, Section 3.3, Section 3.4, Section 3.5, Section 3.6 and Section 3.7, which establishes the central empirical pattern: health co-benefits were reported more frequently, and in richer forms, where climate action involved direct green or blue space contact. Objective 2 builds on this. The CEHN framework was induced from these cross-project regularities and then interpreted through established theory; it therefore rests partly on the present data and partly on the prior literature, and the two should not be conflated. The existence and patterning of psychosocial, behavioural, and structural co-benefits across projects, and their concentration in higher-nature-contact settings, are directly evidenced by the project reports. By contrast, the proposed directionality of the pathways, the moderating roles assigned to community agency and environmental engagement, and the direct biophysical pathway rest more heavily on the prior literature and are not tested here; these are offered as hypotheses for the prospective evaluation described in Section 2.

#### 4.1.1. Core Components

The framework comprises three interconnected components observed across projects. Community agency (CA) was evident through the development of social capital and trust networks, the mobilisation of cultural assets and local knowledge, and the establishment of partnerships with institutions and organisations. Environmental engagement (EE) manifested through the frequency and quality of nature contact, access to ecosystem services such as clean air, water, and green spaces, participation in environmental stewardship activities, and engagement with climate action initiatives. Health outcomes (HOs) included physical health indicators, mental health and psychological wellbeing, social health through community connection, and a sense of purpose.

The observed pathways arise from distinct mechanisms within the core components. It should be noted that neither mediation nor moderation has been formally tested in this study; the terminology “pathway” is used throughout to reflect theoretically grounded associations consistent with moderation effects where one component amplifies or modifies the effect of another, rather than to imply confirmed causal chains. The proposed directionality draws on the established literature for nature-based health interventions [12,27,28,30] and community health mechanisms [17,23,25,26] rather than being derived from the project data alone.

#### 4.1.2. Observed Pathways Linking Community Action to Health

Four observed pathways emerged from the project data, connecting community environmental action to health outcomes (Figure 2).

Within this framework, the community-led climate activities (workshops, stewardship sessions, advocacy events, infrastructure development) constitute the actions; participants reached, events delivered, and community assets created are the outputs. The health and wellbeing changes reported by participants are the outcomes, and represent the co-benefits examined in this study: they arise as secondary, unintended consequences of climate-primary action rather than as programme objectives. The specific co-benefits fall into four categories corresponding to the pathways set out below: (i) direct biophysical co-benefits, including stress relief and physiological restoration, increased physical activity through active travel and outdoor movement, exposure to fresh air, and sensory engagement with natural environments; (ii) psychosocial co-benefits, including improved mental wellbeing, reduced loneliness and social isolation, increased sense of community connection and belonging, friendship formation, identity affirmation among marginalised groups, reduced school refusal and emotional distress in children, place attachment, and constructive management of climate anxiety; (iii) behavioural co-benefits, including uptake of active travel, adoption of growing and food-sovereignty practices, skill development in horticulture and bike maintenance, climate-conscious lifestyle pledges, and intergenerational knowledge transfer; and (iv) structural co-benefits, including expanded community assets (tool libraries, cargo bike schemes, solar installations, seed libraries, accessible mobility equipment), strengthened organisational partnerships and volunteer networks, capacity for civic advocacy, and pathways into employment in community organising. The framework also anticipates longer-term impacts (sustained reductions in health inequalities, strengthened structural determinants of health) but these extend beyond what this retrospective analysis can confirm. We note that these four pathways occupy different positions along the results chain, from enabling conditions through intermediate levers to final health outcomes, and are not intended as a single linear sequence. Consistent with the systems-thinking orientation of the framework, they represent parallel and interacting routes through which community agency and environmental engagement produce health outcomes, rather than discrete stages of a linear logic model. Some elements function simultaneously as co-benefits and as enabling conditions for other pathways: structural changes such as cargo bike libraries, tool-lending schemes, and solar installations are themselves health-relevant outcomes in the social-determinants sense [23], while also creating the material conditions that enable the direct biophysical and psychosocial pathways, a dual role represented by the feedback loops in Figure 2. Likewise, behavioural changes (e.g., active-travel uptake) are intermediate behavioural co-benefits that may, in turn, sustain the ultimate health outcomes (physical and mental health, social connection, personal development, and sense of purpose) that are the co-benefits of primary interest.

Pathway 1: direct biophysical benefits emerge exclusively from environmental engagement, as tangible health improvements result from direct environmental exposure—e.g., microbial benefits, air quality enhancements, physical activity from outdoor movement, and thermal regulation from natural cooling. Participants across multiple projects described experiences of stress relief and restoration, with phrases like “*fresh air on my face*” and “*working outside*” appearing repeatedly. Seasonal outdoor engagement provided opportunities for physical movement and sensory experiences in natural settings.

Pathway 2: psychosocial benefits operate as a moderation effect. Environmental engagement (EE) amplifies and modifies the psychosocial health benefits generated by community agency (CA). Community organising builds social connection, collective efficacy, and shared purpose [25,26], whilst nature contact provides additional psychological restoration and place-based belonging [12,13,27,36], moderating the strength of these outcomes beyond what community action alone would produce. For example, LGBT Health and Wellbeing participants felt more connected to their community and less isolated. ELREC’s workshops facilitated “*long-term friendships*” that reduced loneliness, and Earth in Common participants valued “*meeting new people and working outside*”.

Pathway 3: behavioural change benefits represent a synergistic moderation. Community agency and environmental engagement jointly produce health-promoting behavioural outcomes that neither component generates independently. Environmental engagement provides embodied environmental awareness and motivation, while community agency provides the social learning context, peer norms, and collective accountability that convert awareness into sustained behaviour change [37]. Research on pro-environmental behaviour supports the view that social context moderates the relationship between environmental contact and behaviour change [37,38].

This was evident through skill development in growing, seed saving, and composting, with participants implementing learning in their own gardens, choosing active travel, and making climate-conscious pledges about protecting people and planet.

Pathway 4: structural transformation benefits constitute health co-benefits in the sense used in the literature on social innovation and community-led systemic transitions [39,40]. By reshaping the material, institutional, and social conditions in which health is produced, structural changes generate sustained, population-level health outcomes that extend beyond the individual programme participants. This pathway emerges from community agency through collective organising, advocacy, and mobilisation. Across the projects, this was evident through infrastructure improvements (solar installations, cargo bike libraries, tool lending systems), policy advocacy exemplified by Parents for Future Scotland’s City Chambers event with 74 children and 15 councillors, and institutional capacity building that created lasting community assets beyond individual projects.

#### 4.1.3. Reinforcing Feedback Mechanisms

Two feedback loops sustain and amplify the system over time. Surface Reinforcement (HO ↔ CA) occurs when communities experience tangible health improvements from their collective environmental action. These outcomes strengthen collective efficacy by demonstrating that community-led initiatives produce real benefits, validate the organising approach, and motivate more ambitious action. This was evident across projects where positive health outcomes directly led to sustained community engagement and, in one case, participants transitioning into professional community organising roles.

Adaptive Learning (HO ↔ EE) occurs when individuals experience wellbeing benefits from environmental engagement, creating a virtuous cycle. Direct experiences of health improvement deepen emotional connections to nature, reinforce positive associations with green and blue spaces, and motivate more frequent and sustained environmental engagement patterns. As one Bikes for Refugees participant described, experiencing mental health benefits from cycling through green routes led to daily cycling routines: “*When I have a bad time, I use the bike to go out to get some fresh air and refresh my mind*”.

### 4.2. Contextual Adaptation

Projects showed capacity to adapt to local conditions across diverse environmental, cultural, and socioeconomic contexts, consistent with accounts of adaptive capacity in community-led initiatives [39,40,41]. Environmental adaptation varied according to available natural resources. High-green-space settings emphasised programming of existing natural assets (e.g., Meadows Community Garden, Drylaw orchards), whilst low-green-space contexts required intensive programming to maximise limited natural areas (e.g., Leith Community Croft, community gardens in Wester Hailes and Oxgangs).

Cultural adaptation was evident across diverse populations, for example, ELREC’s workshops served participants from ethnic minority backgrounds, creating spaces where multicultural communities could engage with environmental action through culturally relevant activities like textile upcycling and rag doll making. LGBT Health and Wellbeing created inclusive nature access specifically for LGBTQ+ community members, with one participant noting how “observing beauty and the breadth of queerness in nature” affirmed their identity. Immigrant communities utilised environmental initiatives as platforms for cultural bridge-building and integration, with Bikes for Refugees enabling access to Edinburgh’s green networks while supporting social connection.

Socioeconomic adaptation addressed varying resource availability and access barriers. Projects in resource-constrained contexts integrated environmental programming with basic needs provision, for example, Tummies Not Trash combined food rescue with cooking classes and school outreach. Economic barriers were addressed through tiered pricing (Earth in Common’s concession tickets and free Ostara (spring equinox celebration)) and free events that ensured cost would not prevent participation. Projects also addressed non-financial barriers such as Wee Spoke Hub’s specialised wheelchair handcycle repair services, which supported disabled people’s independence and active travel. The contextual variation observed here aligns with accounts of grassroots innovations for sustainability [41], which emphasise that community-based initiatives succeed precisely because they adapt to local social, economic, and environmental conditions in ways that top-down programmes cannot replicate.

## 5. Discussion

This retrospective analysis suggests that green and blue space engagement may be a key contextual moderator in generating health co-benefits from climate action. Projects with high nature contact showed substantially higher rates of spontaneous positive health outcome reporting compared to low-nature-contact projects. It is important to note that the health outcomes observed were primarily reported as subjective wellbeing, social connection, and behavioural improvements; objective biophysical health data (clinical measurements, validated psychometric instruments, physiological indicators) were not collected in this study. The biophysical pathway in the CEHN framework is therefore theoretically grounded in the existing literature [12,30,42] rather than confirmed by project data, and should be interpreted accordingly. These findings align with established evidence on nature’s restorative effects [12,27,28] and recent work demonstrating health co-benefits from climate mitigation interventions [5,6], suggesting that natural settings may amplify the health-generating potential of community climate action.

Several pathways appeared to link green and blue space climate action to health outcomes. Participants consistently reported mental restoration experiences, such as feeling “*topped up*,” “*refreshed*,” and experiencing stress relief, consistent with attention restoration theory [27,28] and stress reduction theory [29,30]. The facilitating effect of outdoor settings on social bonding aligns with research showing that natural environments promote pro-social behaviour and community cohesion [13,36]. The deeper emotional engagement with climate issues observed in nature-based projects supports emerging evidence that embodied environmental experiences may be more effective than purely informational approaches for fostering pro-environmental behaviour [37].

The finding that setting appeared more strongly related to health outcomes than programme duration has important implications for intervention design. Even brief nature contact outperformed longer indoor programmes, suggesting that natural environments provide inherent health-promoting qualities that enhance climate programming outcomes [12,42]. While longer engagement was associated with higher rates of health outcome reporting within nature-based interventions, consistent with research showing cumulative benefits of nature exposure [4,38], the finding that brief outdoor contact wellbeing benefits exceeded prolonged indoor programming indicates that the setting may be a primary determinant of health co-benefits.

This finding contrasts with typical public health intervention design approaches that have historically treated environmental context as a backdrop rather than as integral to intervention outcomes [43] and merits further investigation.

Blue spaces appeared to offer unique benefits for community climate action. The Water of Leith and Union Canal projects demonstrated that waterways support both active engagement through restoration activities and contemplative connection through creative exercises. While green space health benefits are well-established [12], blue space research remains an emerging field [44,45]. Edinburgh’s extensive blue space network appears underutilised for community climate-health programming and represents an opportunity for expanded intervention development.

The success of culturally responsive programming such as ELREC’s multicultural workshops and LGBT Health and Wellbeing’s inclusive gardening aligns with community-based participatory research principles emphasising the importance of tailoring interventions to specific community needs and strengths [17,18]. These findings suggest that targeted nature-based climate action may effectively reach populations who face barriers to mainstream green space recreation, potentially addressing health equity concerns [46,47].

The observation that nature-based climate projects helped participants manage eco-anxiety and build hope has important mental health implications. Rather than increasing climate distress, hands-on environmental action in natural settings appeared to provide agency and connection that reduced anxiety. This aligns with recent research suggesting that active environmental engagement may buffer against climate-related psychological distress [48,49], contrasting with purely educational approaches that may inadvertently increase anxiety without providing coping mechanisms.

Furthermore, nature-based climate programmes may effectively complement traditional health services, particularly for addressing mental health challenges and social isolation, indicating potential opportunities for cross-sector collaboration between environmental and health service providers.

The findings suggest that green and blue spaces may enhance the health-generating potential of climate action through multiple pathways. Natural settings appear to provide cognitive restoration and stress recovery [27,28,29,30], whilst facilitating social interaction more effectively than indoor environments [13,36]. Direct contact with natural elements creates connections to environmental systems that may enhance both climate motivation and psychological wellbeing, whilst regular engagement in local green and blue spaces builds place attachment [50,51,52] that may benefit both environmental stewardship and mental health.

The CEHN framework provides an understanding of how these health co-benefits emerge. As set out in Section 4.1, the CEHN framework emerged inductively from cross-project patterns and was grounded in established theoretical frameworks—including salutogenesis [22], social–ecological theory [23,24], attention restoration theory [27,28,29,30], and the biophilia hypothesis [31,32]. It should be understood as a hypothesis-generating framework warranting prospective testing rather than a confirmed explanatory account.

Of these four pathways, the direct biophysical pathway warrants a specific caveat. The explicit outcomes available to us are participants’ own descriptions and facilitators’ observations: accounts of outdoor physical activity and active travel, together with reported experiences of stress relief and restoration (e.g., “fresh air on my face”, feeling “refreshed” or “topped up”). The mechanisms through which a biophysical pathway might operate, such as increased physical activity, improved air quality, microbial exposure, and thermal regulation, are inferred from the wider literature [7,12,42] rather than confirmed here, as the project reports contained no clinical or objective measurement. We further note that stress relief and restoration are psychophysiological outcomes that cannot be attributed cleanly to this pathway. They might arise through biophysical mechanisms (e.g., physical exertion or direct sensory contact with nature, consistent with attention restoration and stress reduction theory [27,28,29,30]), or through psychosocial mechanisms such as social connection and shared purpose, and our data cannot distinguish between them. The inclusion of a biophysical pathway in the CEHN framework is therefore the most provisional of the four, grounded in established mechanisms from the literature [12,30] rather than direct measurement from these projects, and should be understood as a theoretically informed proposition awaiting validation through prospective studies with appropriate measurement instruments.

The framework demonstrated flexibility across Edinburgh’s diverse contexts. Projects were adapted to varying green space availability and cultural contexts, suggesting the framework may be applicable beyond this specific setting. However, the framework’s generalisability requires testing in other urban contexts with different environmental and socioeconomic characteristics. The CEHN framework is therefore presented as a starting point rather than a definitive contribution: it is grounded in both the patterns identified across the 32 projects and existing frameworks from environmental psychology and public health [12,27,28], and will be subject to prospective testing in ongoing work in which baseline and follow-up data and direct measures of health and wellbeing are employed directly.

This framework contributes to understanding nature-based climate interventions in three ways. First, it offers potential mechanisms explaining why such interventions may generate health co-benefits. Second, it integrates perspectives from green space and health research [12], climate action psychology [49], and community development [17]. Third, it suggests that both community participation and environmental contact appear important for generating health co-benefits, with some evidence that disadvantaged populations may benefit substantially [46,53].

The observed mental health outcomes align with growing evidence that nature-based interventions can support mental wellbeing [14] and suggest potential for integration with mental health services. However, systematic evaluation would be needed to assess economic implications and healthcare utilisation effects. Investment frameworks for green infrastructure should account for potential health co-benefits alongside environmental returns [54,55,56], though quantifying these benefits remains methodologically challenging.

Previous participatory research has demonstrated the value of community researcher models in urban green and blue space programmes [16]. The Edible Estates project’s approach of training and employing local residents extends this model to climate adaptation work, suggesting potential for programme designs that combine climate action with community development. The capacity building observed across projects suggests that investment in nature-based climate action may build lasting community assets that continue to generate benefits beyond funding periods.

The findings have several potential implications for policy and practice, though these require further investigation. The apparent health co-benefits of nature-based climate interventions suggest that funding mechanisms could consider both environmental and health outcomes when evaluating community climate projects [5]. The success of projects integrating green and blue space access with social connection indicates potential value in cross-sectoral approaches that address environmental and health objectives together [6]. However, systematic evaluation would be needed to assess scalability and cost-effectiveness.

The success of projects serving marginalised populations is consistent with evidence that nature-based interventions may help address health inequalities [46,47], though targeted research would be needed to understand mechanisms, effectiveness and optimal approaches for different populations. Integration with existing frameworks such as social prescribing [57], and specifically green social prescribing, which links participants to nature-based community activities as part of non-clinical health support, represents a potential implementation pathway, particularly given growing policy interest in nature-based health interventions.

### Study Limitations and Future Research Directions

This retrospective analysis relied on spontaneous reporting by project implementers rather than systematic and longitudinal health measurement using validated instruments, introducing several limitations. Organisations may have selectively highlighted positive outcomes whilst minimising challenges; we cannot distinguish between genuine health improvements and programme satisfaction; causal relationships between specific programme elements (nature contact versus social interaction versus skill development) cannot be established; and most outcomes represent immediate responses rather than sustained wellbeing changes. However, the consistency of nature-related wellbeing themes across diverse projects strengthens confidence in the findings. Generalisability is further limited by potential selection bias, as organisations applying for climate funding may be predisposed toward holistic approaches that generate health co-benefits. Additionally, Edinburgh’s particular green space assets, cultural attitudes toward nature, and community sector capacity may limit transferability to other contexts.

A further important limitation concerns the analytical value of working across 32 retrospective project reports. This approach provides breadth of pattern recognition across diverse project types, but does so at the cost of depth: the data cannot support detailed analysis of the mechanisms by which nature contact translates into health outcomes, and does not permit the examination of causal pathways through in situ observation or participant interviews. A more mechanistically informative approach would involve narrowing the focus to a smaller number of purposively selected cases, for example, three to five projects differing systematically in scale, nature contact level, and community context, and applying mixed methods—combining participant interviews, structured observation, and validated wellbeing instruments—to examine how health co-benefits arise within those settings. Such an approach would allow the CEHN pathways to be examined at the level of individual participant experience and social process, rather than inferred from aggregate reporting patterns. The present study should therefore be understood as a first-stage, hypothesis-generating analysis: its contribution is to establish the existence of a cross-project pattern and to propose a theoretical framework to explain it, rather than to confirm that framework or to describe the mechanisms with granularity. In-depth, prospective case studies are a natural and important next step, and are already underway as part of the ongoing GroundsWell evaluation programme.

The absence of wellbeing reporting from some projects does not necessarily reflect weaker performance. All projects met their stated climate action goals. Projects reporting fewer health co-benefits were often highly successful infrastructure projects that achieved their environmental objectives whilst generating valuable social connections and skill development. Variation in wellbeing reporting likely reflects differences in the project setting and reporting focus rather than programme quality.

Future research should employ prospective designs that operationalise the CEHN framework through community-led monitoring systems. Training project participants to document their own wellbeing changes as part of the evaluation process could provide more comprehensive and authentic outcome data whilst capturing the dynamic interactions between community agency, environmental engagement, and health outcomes in real time [16,58,59]. Such approaches would allow for context-specific adaptation across diverse settings whilst maintaining rigorous assessment of health co-benefits.

Specific research priorities include: (1) longitudinal studies examining sustained health outcomes and climate engagement; (2) studies examining the pathways through which community-led climate action involving nature contact produces health benefits, including optimal programme design elements and how active climate engagement, nature exposure, and social connection interact, ideally through in-depth case study designs incorporating validated wellbeing instruments, structured observation, and longitudinal follow-up; (3) population-specific research exploring cultural adaptation and effectiveness across diverse health conditions; and (4) implementation science investigating community readiness and institutional integration factors for nature-based climate interventions.

## 6. Conclusions

The retrospective nature of this analysis highlights the value of examining real-world community initiatives to understand how environmental and health outcomes interact. While these projects were designed to address climate challenges, many demonstrated significant health co-benefits, particularly when involving green and blue space engagement, pointing to synergistic relationships between community action, environmental engagement, and health outcomes that warrant theoretical examination.

Using the GreenLight projects as case studies, we develop the Community–Environment–Health Nexus (CEHN) framework—a framework explaining how community-led environmental action in natural settings generates health co-benefits through interconnected pathways of community agency, environmental engagement, and health outcomes, with practical guidance for designing interventions that maximise co-benefits across diverse settings.

Projects with high nature contact showed nearly four times higher rates of health outcome reporting compared to built environment projects, with extended engagement producing substantially stronger benefits, particularly for structurally disadvantaged populations. Rather than increasing climate anxiety, hands-on environmental engagement helped participants build hope and resilience, whilst natural settings enhanced social bonding across the vast majority of projects.

These findings point toward potentially productive synergies between environmental and health objectives when community-led climate action incorporates meaningful green and blue space engagement. The CEHN framework offers a preliminary, inductively derived framework for understanding how such co-benefits may arise, one that requires prospective empirical testing before stronger claims can be made. Edinburgh’s experience provides an empirically informed starting point for such investigation, though the scope and generalisability of these findings will depend on the results of ongoing research.

## Figures and Tables

**Table 1:** Complete analysis of GreenLight projects by nature contact and health outcomes.

Project Name	Nature Contact Level	Health Outcomes Reported	Specific Health Outcomes
Earth in Common	High	Yes	Mental wellbeing, social connection (4.93/5 satisfaction), * 90.2% enjoyed social aspects, 75.6% improved environmental knowledge
Youth Vision	High	Yes	Confidence building, behavioural change, teachers noted reduced crying/school refusal, friendship formation
The Green Team (Frogston)	High	Yes	Teacher-rated wellbeing improvements (6/7), enhanced confidence, social skills development (5/7)
LGBT Health & Wellbeing	High	Yes	* 76% mental wellbeing improvement, 88% felt more connected to community, 53% felt less isolated
Drylaw & Telford Community Association	High	Yes	Intergenerational learning, climate awareness, community engagement with 36 children + adults
Edible Estates	High	Yes	Community capacity building, 1 person gained employment, expanded social networks through 22 partnerships
The Meaning Map Project	High	Yes	Participants felt “topped up,” “nourishing,” community connection, reduced feelings of powerlessness
Bridgend Farmhouse	High	Yes	Community connection, positive family experiences: “my kids had a ball,” “such a nice friendly event”
Transition Edinburgh South	High	Yes	Inclusion benefits for LGBT+ community, space for stigma-free participation
Meadows Community Garden	High	No	No health outcomes specifically reported in available documentation
Bikes for Refugees	Moderate	Yes	Mental health benefits, stress relief, independence, reduced loneliness, “fresh air on my face”
BANZAI Cargo Bike Library	Moderate	Yes	Family safety/wellbeing, independence, reduced transport anxiety, community confidence
Parents for Future Scotland	Moderate	Yes	* 96.4% learned something new, 82.3% more likely to use active travel, community efficacy building
Porty Community Energy	Moderate	Yes	Community engagement, confidence in sustainable choices, inspiration and hope
ELREC—Conscious Living	Moderate	Yes	Mental wellbeing through “relaxed and friendly environment,” community building, skill-sharing
ELREC—Open Arms	Moderate	Yes	Friendship formation, reduced loneliness and isolation, mental health benefits
Edinburgh Tool Library—Cycle Kitchen	Moderate	Yes	Members got “back on the road,” reduced reliance on motorised transport, skill confidence
Edinburgh Old Town Development Trust	Moderate	Yes	Community connections, volunteering opportunities, skill development for 30+ people
Grow2Eat/Settlement Projects	Moderate	Yes	Connection to natural cycles, accessibility for people without gardens, community engagement
SCCAN/1000 Better Stories	Moderate	Yes	Intergenerational dialogue, climate adaptation awareness, family engagement
Salisbury Centre	Moderate	Yes	Skills confidence, participants implemented learning in own gardens, enthusiasm for growing
The Green Team (e-cargo bike)	Moderate	Limited	Enhanced ability to deliver programmes but no direct health outcomes reported
Rhyze Mushrooms	Moderate	No	Focused on production scaling—no health outcomes reported
Broomhouse Community Growers	Moderate	No	Environmental benefits noted but no health outcomes reported
Wee Spoke Hub	Low	Yes	Empowerment for wheelchair users, independence, “hugely empowering devices”
ESALA University of Edinburgh	Low	Yes	Hope vs anxiety balance, networking, enthusiasm, connection between industry parts
Edinburgh Tool Library	Low	Limited	Some social connection mentioned but minimal wellbeing details
EdinBRIC	Low	Limited	Community building mentioned but limited wellbeing details
QPC Homes (Cozy Futures)	Low	Limited	Residents expressed gratitude for cost savings but minimal health focus
Eala Impacts (Reuse Rover)	Low	No	No specific health outcomes reported in available documentation
Marco Tenconi/Rhyze	Low	No	Infrastructure project—no health outcomes reported
Tummies Not Trash	Low	No	No specific health outcomes reported in available documentation

* Percentages represent the proportion of participants reporting each outcome.

**Table 2:** Engagement duration–outcome analysis (all projects categorised).

Engagement Duration Category	Number of Projects	Health Outcome Reporting Rate (Projects)	Key Characteristics
Extended (20+ contact hours across multiple sessions over 8+ weeks)	3	100% (3)	Deep nature immersion, sustained behavioural change, lasting environmental connection
Moderate (4–20 contact hours, whether delivered as intensive full-day sessions or spread across 2–7 weeks)	10	90% (9)	Meaningful green/blue space engagement, community building through nature
Brief (1–4 contact hours delivered in 1–2 sessions)	6	83% (5)	Positive nature experiences, immediate environmental connection
No green/blue space	13	38% (5)	Indoor/infrastructure focus, limited nature-mediated benefits

## Data Availability

The data analysed in this study consist of final project reports submitted to the Edinburgh Communities Climate Action Network (ECCAN) by GreenLight programme recipients (2024–2025). The reports are not publicly available as some were shared under organisational consent, and the anonymised coded dataset is not available due to privacy and ethical restrictions.

## Abbreviations

The following abbreviations are used in this manuscript:

CA	Community Agency
CEHN	Community–Environment–Health Nexus
CIC	Community Interest Company
ECCAN	Edinburgh Communities Climate Action Network
EdinBRIC	Edinburgh Building Retrofit Collective
EE	Environmental Engagement
ELREC	Edinburgh and Lothians Regional Equality Council
ESALA	Edinburgh School of Architecture and Landscape Architecture
HO	Health Outcomes
LGBT	Lesbian, Gay, Bisexual, and Transgender
LGBTQ+	Lesbian, Gay, Bisexual, Transgender, and Queer/Questioning+
SCCAN	Scottish Communities Climate Action Network
WHO	World Health Organization

## Appendix A

Based on the documents provided, here are the 32 GreenLight projects with brief descriptions:

### GreenLight Projects 2024–2025

1. Edible Estates—Developed community-led climate adaptation plans across four Edinburgh council estates (Hutchison, Lochend/Craigentinny, Wester Hailes, and Oxgangs), training local community researchers to understand climate risks and develop practical, neighbourhood-specific adaptation strategies.
2. Earth in Common—Created a new plant nursery at Leith Community Croft and delivered a series of green skills workshops including carbon literacy sessions, nature connection activities, bike maintenance, foraging, and climate-themed community events throughout autumn and winter.
3. Porty Community Energy—Travel Agents of Change—Ran a creative campaign collecting over 60 stories of flight-free holidays, producing an illustrated zine and touring exhibition across Edinburgh to promote sustainable travel choices and spark conversations about low-carbon tourism.
4. Edinburgh Tool Library—Opened a new satellite library location in Granton (pivoting from the originally planned Wester Hailes site), expanding their tool lending services to reduce consumption and support the circular economy in underserved areas.
5. BANZAI Cargo Bike Library—Established a community cargo bike library in Bruntsfield, purchasing cargo bikes and setting up a lending system to promote active travel and provide sustainable transport options for families and local residents.
6. Tummies Not Trash—Created a zero-food-waste hub in Gorgie, rescuing surplus food from supermarkets and redistributing it through community partnerships while running cooking classes and educational outreach to schools and community groups.
7. Bikes for Refugees Scotland—Repaired and distributed 76 bikes directly to refugees and asylum seekers across Edinburgh, providing sustainable transport, supporting access to services, and promoting mental wellbeing and community integration.
8. The Meaning Map Project—Conducted participatory arts workshops across Edinburgh where residents mapped their emotional relationships to local places, creating “meaning maps” that sparked climate conversations rooted in memory, hope, and place-based connection.
9. Rhyze Mushrooms Cooperative—Installed bespoke shelving infrastructure in their polytunnel at Lauriston Farm to scale up sustainable mushroom production, increase use of recycled business waste as growing substrate, and expand community education opportunities.
10. Drylaw and Telford Community Association—Restored two community fruit orchards through intergenerational volunteering sessions, teaching pruning and grafting skills while engaging local children and families in practical food growing and biodiversity conservation.
11. EALA Impacts CIC—Developed the “Reuse Rover” concept (a mobile shipping container) to collect reusable construction materials from demolition sites for redistribution to social enterprises, though the project was delayed due to venue changes.
12. Youth Vision—Delivered experiential outdoor learning programmes for disadvantaged young people aged 16+, focusing on edible woodland restoration, biodiversity enhancement, traditional building skills, and creating accessible pathways through their farm steading.
13. Broomhouse Community Growers Association—Installed solar power systems and switched from petrol to battery-powered garden equipment, reducing noise pollution and environmental impact while providing sustainable energy for their community growing activities.
14. Wee Spoke Hub (SHRUB Coop)—Provided specialised repair services for wheelchair handcycles and mobility equipment, addressing a significant gap in accessible transport maintenance and supporting disabled people’s independence and active travel.
15. Bridgend Farmhouse—Organised apple pressing and tree planting community events, saving 250 kg of apples from waste to produce 110 bottles of juice while engaging 266 local residents in sustainable food practices and environmental stewardship.
16. Salisbury Centre—Established a new seed library at Gracemount Community Walled Garden in partnership with their existing seed library, running workshops to teach seed saving skills and promote plant sovereignty in local communities.
17. Parents for Future Scotland—Delivered air pollution education programmes across 20 Edinburgh schools, reaching 2635 children and 132 parents, culminating in a City Chambers advocacy event where pupils presented demands for cleaner air and safer streets.
18. Edinburgh Building Retrofit Collective (EdinBRIC)—Continued community outreach on building retrofit through their mobile “Retrofit Rover,” supporting volunteer training and attending community events to raise awareness about home energy efficiency and decarbonization.
19. The Green Team (Cargo Bike)—Purchased an electric cargo bike to expand their Green Schools programmes, enabling more efficient delivery of environmental education to schools across Edinburgh while demonstrating sustainable transport.
20. Edinburgh Old Town Development Trust—Launched a community growing collective to create and enhance garden spaces across Edinburgh’s Old Town, establishing partnerships and organising workshops to support food security and biodiversity in the city centre.
21. Settlement Community Project/Grow2Eat—Set up regular growing stalls on Leith Walk providing free seeds, compost, and growing guidance to promote urban food growing, particularly targeting people without gardens and running foraging walks.
22. Transition Edinburgh South—Supported their Queer Gardening Group at Gracemount Walled Garden, creating inclusive spaces for LGBTQ+ community members to engage in growing, seed saving, and climate action through regular gardening sessions.
23. ELREC Conscious Living Project—Delivered sustainable rug weaving workshops for 54 participants from diverse ethnic backgrounds, repurposing over 9 kg of fabric waste while fostering community connections and promoting sustainable textile practices.
24. ELREC Open Arms—Ran rag doll making workshops for minority and migrant women using recycled materials, creating 32 dolls while facilitating climate conversations and supporting mental wellbeing through creative community activities.
25. The Green Team (Frogston Research)—Collaborated with university researchers to study how children engage with environmental stewardship, working with two groups of eight children each over 13 weeks to understand learning outcomes from nature-based activities.
26. LGBT Health and Wellbeing—Hosted a community-led “Learn to Grow” workshop for 20 LGBTQ+ participants, combining horticultural education with nature connection and wellbeing activities to create inclusive climate action spaces.
27. Edinburgh Tool Library Cycle Kitchen—Provided bike maintenance training for two additional volunteers to expand their community bike repair services, helping 218 participants maintain their sustainable transport and reduce reliance on cars.
28. University of Edinburgh ESALA—Organised the “Back of the Envelope” workshop for 34 architects and students, facilitating discussions on innovative data approaches for climate-aware architectural design and sustainable building practices.
29. QPC Homes (Cozy Future)—Built community engagement infrastructure within a tenement building to address maintenance issues and lay the groundwork for future clean energy upgrades, establishing communication systems and resident networks.
30. Meadows Community Garden—Purchased tools and tree stakes to enhance biodiversity through composting system improvements and native hedgerow development, supporting weekly volunteer activities in this urban green space.
31. SCCAN (Stories Project)—Organised a family storytelling workshop called “The Most Beautiful Place in the World” at Stockbridge Library, using mapping and creative activities to engage parents and children in climate conversations.
32. Marco Tenconi—Working with Rhyze Mushrooms Cooperative project, focusing on mushroom growing infrastructure development.
